# Editorial: Nutraceuticals modulation for oxidative stress in disease and health: volume II

**DOI:** 10.3389/fphar.2026.1971692

**Published:** 2026-09-07

**Authors:** Kok-Yong Chin, Kok-Lun Pang, Chun-Wai Mai, Alessandro Salatino

**Affiliations:** 1 Department of Pharmacology, Faculty of Medicine, University Kebangsaan Malaysia, Kuala Lumpur, Malaysia; 2 Jeffrey Cheah School of Medicine and Health Sciences, Monash University Malaysia, Subang Jaya, Selangor, Malaysia; 3 Centre for Cancer and Stem Cell Research, Institute for Research, Development and Innovation (IRDI), IMU University, Kuala Lumpur, Malaysia; 4 Dana-Farber Cancer Institution - Harvard Cancer Centre, Boston, MA, United States

**Keywords:** anti-inflammatory, antioxidants, free radical, natural products, plant bioactives

Oxidative stress and inflammation are two primary pathological engines driving the global burden of chronic diseases. Together, they operate as a self-amplifying loop, whereby reactive oxygen species (ROS) cause tissue damage and trigger chronic inflammation. The recruitment of immune cells and the release of ROS exacerbate tissue damage. In addition, these two processes are linked by the key molecular switches, i.e., nuclear factor kappa B (NF-κB) for inflammation, and nuclear factor erythroid 2-related factor 2 (Nrf2) for antioxidant defence. Their relationship is bidirectional, and the net effects are context dependent (Altanam et al., 2025). This specific inflammation-oxidative stress axis offers an avenue for designing interventions to prevent and manage disease. The 11 articles curated for this Research Topic serve as a bridge between fundamental molecular discovery and population-level health. These studies demonstrate that nutraceuticals are not merely radical scavengers but signalling modulators capable of interrupting the cycle of chronic cellular damage.

Organ-specific preclinical models serve as the foundation for identifying the precise mechanisms of nutraceutical action against disease, thereby providing preliminary evidence to support the transition to human trials. For instance, the study by Li et al. on *Achyranthes bidentata* polysaccharides (ABPS) represents a multi-targeted approach to reducing hypoxic renal injury. By decreasing the formation of neutrophil extracellular traps, which are complexes of DNA and proteins that amplify tissue damage, and by suppressing the NOD-like receptor family pyrin domain-containing 3/apoptosis-associated speck-like protein containing a CARD/caspase-1 signalling pathway, the ABPS group achieved significant functional recovery in the study. In another renal model, Aati et al. demonstrate that Saudi Tamarix honey mitigates cisplatin-induced nephrotoxicity by modulating the signal transducer and activator of transcription 3/Nrf2 signalling axis. The scoping review by Wang et al. provide a comprehensive evaluation of *Eucommia ulmoides*, reinforcing its role in maintaining skeletal resiliency through the modulation of key bone signalling pathways, such as bone morphogenetic protein/suppressor of mothers against decapentaplegic, Wnt/β-catenin, osteoprotegerin/receptor activator of nuclear factor kappa-B ligand, and mitogen-activated protein kinase/NF-κB.

Beyond the kidneys, Taysi et al. investigate the efficacy of quercetin in providing cardiac protection following traumatic injury. Zhan et al. establish the role of ginkgolide B as a regulator of retinal health via the AMP-activated protein kinase/Nrf2 pathway. Since the retinal pigment epithelium is vulnerable due to its constant exposure to light-induced photo-oxidation and high metabolic demand, Nrf2 activation promotes ocular longevity by upregulating endogenous defence systems, rather than merely scavenging existing radicals. On the other hand, Essa et al. establish a protective role for taurine against microplastic-induced splenic toxicity, addressing an emerging environmental threat to inflammatory homeostasis. Collectively, these findings underscore the capacity of isolated bioactive compounds to enhance tissue-specific defences against complex environmental and pathological stressors.

While these preclinical data are robust, the clinical translation of these compounds depends on their pharmacodynamic and pharmacokinetic behaviour in complex human organ systems. The “Valley of Death” in nutraceutical research refers to the gap in which promising preclinical results fail to translate into human efficacy due to poor bioavailability, formulation instability and toxicity concerns. Bridging this gap requires rigorous human exposure data and long-term stability metrics (Fatima et al., 2025). In the trial by Patel et al., a liquid, plant-based liver supplement highlights the importance of therapeutic outcomes and a 24-month shelf life to ensure bioactive potency. Furthermore, the work of Smeriglio et al. analyse citrus flavanone in both plasma and urine, mapping how these compounds are metabolised and cleared, a prerequisite for effective dosing protocols. Understanding these systemic parameters allows the field to move from generalised consumption toward targeted topical and environmental applications of bioactive extracts.

In line with the paradigm shift towards the “Circular Economy,” agricultural byproducts are upcycled into high-value health resources to fulfil both human health and environmental sustainability mandates (Ingallina et al., 2025). Smeriglio et al. present a clear example by using red grape pomace extract (GPE) as a sustainable photoprotective agent. The mechanistic rigour of this study was enhanced by measuring specific biomarkers, including inhibition of albumin denaturation and protease activity, thereby validating its multi-target anti-inflammatory potential. This study demonstrates how agricultural waste can be upcycled into high-value, broad-spectrum skin protection products to counter environmental oxidative stress.

While individual bioactives demonstrate efficacy in preclinical disease models, the ultimate validation of these therapeutics requires a transition from the laboratory to population health. The meta-analysis by Cai et al. provide evidence that dietary nitrate intake improves metabolic outcomes, particularly in weight management. Furthermore, Tian et al. utilise data from the National Health and Nutrition Examination Survey in the USA to investigate the oxidative balance score (OBS), a composite metric comprising 16 dietary and 4 lifestyle factors, in relation to allergic rhinitis (AR). They demonstrate that OBS is more clinically relevant than tracking individual vitamins because it accounts for the complex interplay between pro-oxidants (such as smoking) and antioxidants. The study found a positive association in which higher OBS was associated with higher odds of AR. The authors speculated that this association is attributed to “reverse causation”, in which symptomatic individuals increase their intake of healthy foods. This untested hypothesis requires further validation. Nevertheless, this highlights the common thread between individual dietary patterns and the overarching molecular mechanisms discussed in previous paragraphs.

Overall, this volume synthesises the three essential pillars of modern nutraceutical science: mechanistic rigour in preclinical models, translational validity through pharmacokinetic and clinical assessment, and macroscopic impact through epidemiology. To ensure continuous advancement in the field, future research should prioritise standardisation of extracts to ensure chemical reproducibility to satisfy regulatory and clinical requirements. The microbiota-mediated transformation of nutraceuticals is also the next Frontier, given the emerging role of the gut microbiome in converting bioactives into active systemic metabolites (Zhao et al., 2022). Lastly, longitudinal human trials are required to establish definitive causal links between nutraceutical interventions and long-term health outcomes. The future of personalised medicine may depend on the strategic application of these multi-targeted compounds to maintain the delicate equilibrium between oxidative stress and inflammatory response, ultimately reducing the global burden of chronic disease.
